# Integrating a Geneticist in a Multidisciplinary Clinic for Down Syndrome Increases Commitment to Genetic Counseling

**DOI:** 10.1097/pq9.0000000000000039

**Published:** 2017-08-25

**Authors:** Stephanie L. Santoro, Theodora Jacobson, Stephanie Lemle, Thomas Bartman

**Affiliations:** From the * Division of Genetics, Nationwide Children’s Hospital, Columbus, Ohio; † Institute for Genomic Medicine, Nationwide Children’s Hospital, Columbus, Ohio; ‡ Quality Improvement Services, Nationwide Children’s Hospital, Columbus, Ohio; and § Director of Quality Improvement, Neonatal Services, Nationwide Children’s Hospital, Columbus, Ohio.

## Abstract

**Background:**

Although most physicians and genetic professionals are familiar with Down syndrome, many families do not have experience with Down syndrome before having a child diagnosed. The American Academy of Pediatrics has specific recommendations for genetic counseling and chromosome analysis for Down syndrome.

**Local Problem:**

The literature indicates that adherence to completion of appropriately timed genetic counseling is low at 31%. This study was initiated to determine our adherence rates and to improve if needed.

**Methods:**

In the Down syndrome clinic at Nationwide Children’s Hospital, a subspecialty clinic in the Division of Developmental and Behavioral Pediatrics, a genetic counselor was on-call but did not routinely attend. The intervention consisted of multidisciplinary care with the presence of a clinical geneticist. Statistical Process Control Charts and Fisher’s exact test were used to determine the impact of the intervention.

**Results:**

Our baseline rate of adherence to genetic counseling was similar to previous publications. Direct genetics involvement in the Down syndrome clinic in place of an on-call genetic counselor led to significant improvement in adherence to genetic counseling recommendations over a 6-month period from 35% to 62%, *P* < 0.001 and sustained for 6 months. Postclinic adherence rates and subanalyses by age showed similar results. The final postvisit adherence rate of 89% in February 2017 demonstrates continued improvement. Geneticist involvement allowed chromosome reports uploading and karyotype listing in electronic medical records.

**Implications and Lessons Learned:**

Genetic counseling in newborns with Down syndrome is important, yet was often not received at Nationwide Children’s Hospital before this study. Integrating a geneticist resulted in improvement. Implementing similar models at other institutions can ensure that the correct genetic testing is completed, results documented and families counseled appropriately.

## Abstract 2

Supplemental Digital Content is available in the text.

## INTRODUCTION

Down syndrome has an incidence of approximately 1 in 792 births making it the most common liveborn trisomy and chromosomal condition.^1^ Although Down syndrome may be familiar to most physicians and genetic professionals, many families do not have experience with Down syndrome before having a child diagnosed.^2^ Initial discussions about Down syndrome require health care professionals who are knowledgeable about Down syndrome and who have specific training in delivering sensitive diagnoses.^3^

Chromosome analysis confirms the diagnosis of Down syndrome and enables patient-specific counseling. Accurate diagnostic confirmation with up-to-date information about prognosis provides families with reassurance and leads to acceptance of the diagnosis.^4^ Though Down syndrome is typically due to free trisomy 21, mosaicism and translocations are possible, which may not be well-understood by all.^5^ Importantly, identifying a child with translocation Down syndrome can lead to discussion about parental testing. If a parent is found to have a balanced Robertsonian translocation, recurrence risk can significantly change from < 1% to up to 100% depending on the specific arrangement.^5^ Families should be provided accurate recurrence risk for future family planning and to discuss reproductive options.^5^

In addition to confirmatory chromosomes, there are various practice guidelines and recommendations about how to best deliver a diagnosis of Down syndrome to a family and provide genetic counseling.^3,5–7^ Local genetic counseling adherence rates in our institution were previously unknown. We expected comparability to the published rate of 31% due to similarities between Nationwide Children’s Hospital and Cincinnati Children’s Hospital Medical Center.^8^

We initiated this study to ascertain our genetic counseling adherence rate in children with Down syndrome. As a component of genetic care, we ensured completion of appropriate genetic testing. The purpose of the study was to improve adherence to those 2 recommendations for Down syndrome: genetic counseling and chromosome analysis. Given the multiple barriers to completion of recommended guidelines, including time, knowledge, awareness, and buy-in, we created a key driver diagram with a focus on various stakeholders and phases on care (**see Figure, Supplemental Digital Content 1**, http://links.lww.com/PQ9/A14). We focused on streamlining the process by which families receive genetic counseling and chromosome studies. We expected that a direct geneticist presence in the Down syndrome clinic would decrease the need for families to keep multiple appointments, ease availability of genetics services, and subsequently lead to improved adherence.

## METHODS

This quality improvement initiative involved retrospective review to obtain baseline adherence followed by prospective tracking of patients to follow the impact of intervention. For ease of patient tracking over time, information including demographics was maintained (Table 1). Patients had visits from July 2015 to February 2017 in the Down syndrome subspecialty clinic at Nationwide Children’s Hospital. Nationwide Children’s Hospital is a large free-standing pediatric research center and health care system delivering care to more than 1 million patients each year. The multidisciplinary Down syndrome clinic includes developmental pediatricians and nurse practitioners, psychologists, therapists, and a social worker. Before this quality improvement initiative, an on-call genetic counselor was available if needed.

**Table 1.**
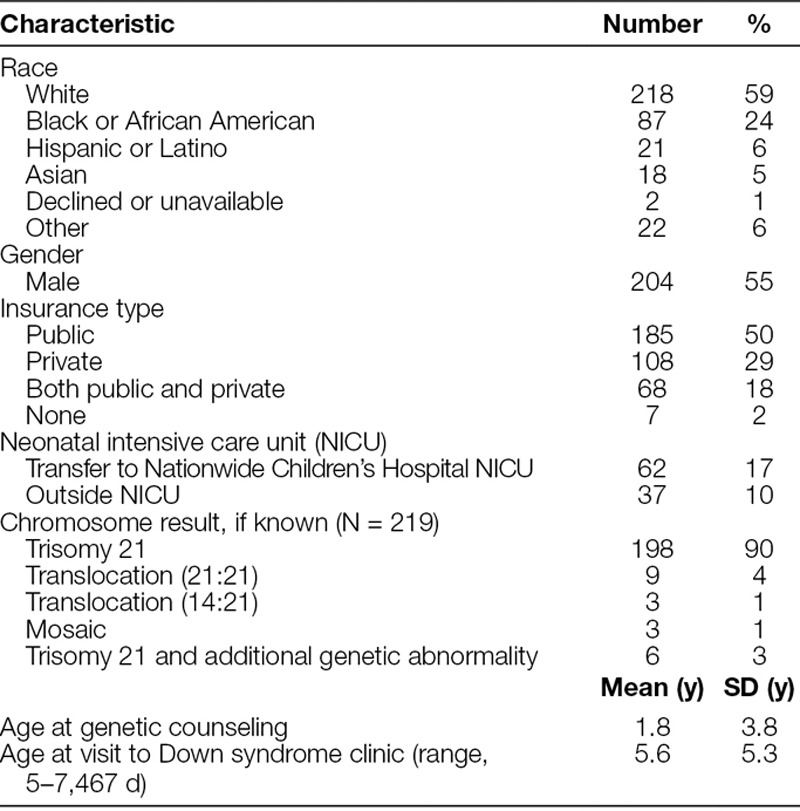
Demographic Information for 368 Patients Scheduled in the Down Syndrome Clinic at Nationwide Children’s Hospital, July 2015 to February 2017

The intervention consisted of incorporating a clinical geneticist into the biweekly Down syndrome multidisciplinary clinic who was available, engaged and capable of completing unmet recommendations if needed. The impact of this clinical change was tracked for 6 months. Before clinic, the geneticist reviewed medical records of scheduled patients including the electronic medical records (EMRs) and records in the cytogenetics laboratory. If applicable, chromosome reports were obtained from the cytogenetics laboratory.

Adherence rates of 2 recommendations, genetic counseling and chromosome analysis, were documented before arrival to the Down syndrome clinic. Adherence to genetic counseling was complete if any of the following were present: a visit with a genetic professional (geneticist or genetic counselor), a progress or consult note from a genetic professional or any identifiable contact with a genetic professional (telephone notes, letters, or pedigrees). If either a chromosome report or a karyotype in a note from a genetics professional were present, adherence to chromosome studies was complete.

For patients without genetic counseling before arrival, the clinical geneticist met with the family following the visit with the developmental pediatrician/nurse practitioner. At the beginning of the discussion, the clinical geneticist asked the family if they had ever met with a genetics professional. If so, genetic counseling was deferred and the patient was considered adherent to genetic counseling at arrival. During the course of genetic counseling, a pedigree and basic intake information was collected from parents. Intake questions included diagnosis details, diagnosis time, and neonatal care. “Prenatal” diagnoses included both screening with cell-free fetal DNA and diagnostic amniocentesis. Rarely, during this process, families would recall meeting with a prenatal genetic counselor—in this case, an abbreviated review of genetic counseling was completed and the patient was considered adherent at arrival.

The Nationwide Children’s Hospital Cytogenetics laboratory receives samples for chromosome studies from many hospitals in Central Ohio. Efforts were taken to search the cytogenetics laboratory database for prior chromosome reports. Additionally, if no chromosome report was found, parents signed medical records releases to allow us to obtain copies of chromosome reports from outside hospital laboratories. If a chromosome report was identified, that patient was considered adherent to chromosome analysis at arrival.

On each clinic day, the number of adherent patients was divided by the number of patients scheduled in the clinic to calculate the adherence rate. To capture overall baseline adherence of the clinic population, we included patients who were scheduled but did not arrive to clinic (no-show patients). Adherence rates per clinic date were calculated (number of patients adherent/number of patients scheduled in Down syndrome clinic), recorded, and tracked. Monthly rates were plotted on p-charts or run charts, and comparisons were made using Fisher’s exact test. Centerline shifts were determined using standard statistical process control chart rules.^9,10^ Postclinic adherence rates, including genetic counseling in the Down syndrome clinic and prior to the time of clinic, were also calculated and tracked monthly. Lastly, we performed age-based subanalysis using the following parameters: (1) age at visit < 12 months; (2) date of birth after 2011 and age ≥ 12 months; and (3) date of birth in 2011 or prior. We chose these parameters based on the importance of genetic counseling shortly after birth and the publication year of the most recent American Academy of Pediatrics guidelines. As a related measure, the age at genetic counseling was followed, with monthly means calculated and tracked over time.

This project was reviewed by our institutional review board chair and identified as quality improvement and was therefore exempt from full institutional review board review. Patient demographic information and aspects of genetic counseling were reviewed for clinical purposes: to determine if patients were in need of meeting with a clinical geneticist for genetic counseling. The purpose of following adherence over time was to determine compliance with standard clinical care per the American Academy of Pediatrics. All data in this article are deidentified, in aggregate format without the ability to link information to an individual patient.

## RESULTS

Of the 667 total appointments scheduled from July 2015 to February 2017, 50 were no-show visits and 617 visits were completed. During this time, some patients returned for follow-up visits. These 617 visits corresponded to 368 distinct individuals who received care in the Down syndrome clinic. Demographic information showed racial diversity, slight male predominance, and various forms of insurance type (Table 1). Within our population, 27% reported admission to a neonatal intensive care unit. For those with a chromosome report available, results showed expected ratios with high rates of free trisomy 21. Of the 9 patients with a specific Robertsonian translocation (21;21), 1 was identified as part of this quality improvement study without previous documentation in the patient’s EMR.

The adherence to chromosome studies differed by source: documentation or parent-report. At baseline, a chromosome report was found in the current EMR for 48 of 368 patients and a karyotype was listed in a geneticist’s note in an additional 71 of 368 patient’s charts. However, many more parents reported that chromosome studies had been completed for their child. To update clinical records, laboratory reports were requested for 127 patients. From these requests, 102 chromosome reports were obtained and uploaded into the current EMR. In total, 227 patients had baseline chromosome studies completed. Genetics involvement resulted in a chromosome laboratory order for only 6 patients.

In total, of the 368 patients seen, 133 patients had genetic counseling before arrival to the Down syndrome clinic (Table 2). Of these, outpatient clinic visits were more common than inpatient hospital consults. Although uncommon, 4% of all patients with a documented genetics referral but no visit due to either the appointment not being scheduled, appointment cancelation, or no-show to a scheduled appointment. The presence of a geneticist in the Down syndrome multidisciplinary clinic resulted in clinical genetic counseling for 114 additional patients. At the conclusion of this study, 247 patients in the clinic had received genetic counseling.

**Table 2.**
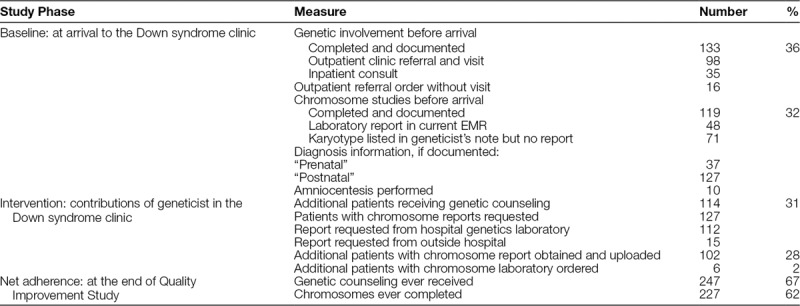
Genetic Counseling and Chromosome Study Adherence Rates in 368 Patients in the Down Syndrome Clinic

**Fig. 1.**
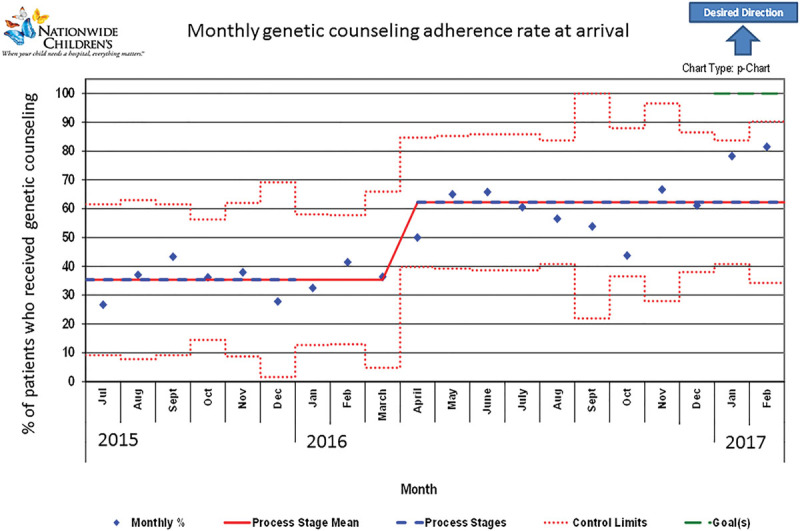
Monthly genetic counseling adherence rate at arrival to a multidisciplinary clinic for Down syndrome; geneticist presence implemented in October 2015 increases adherence.

**Fig. 2.**
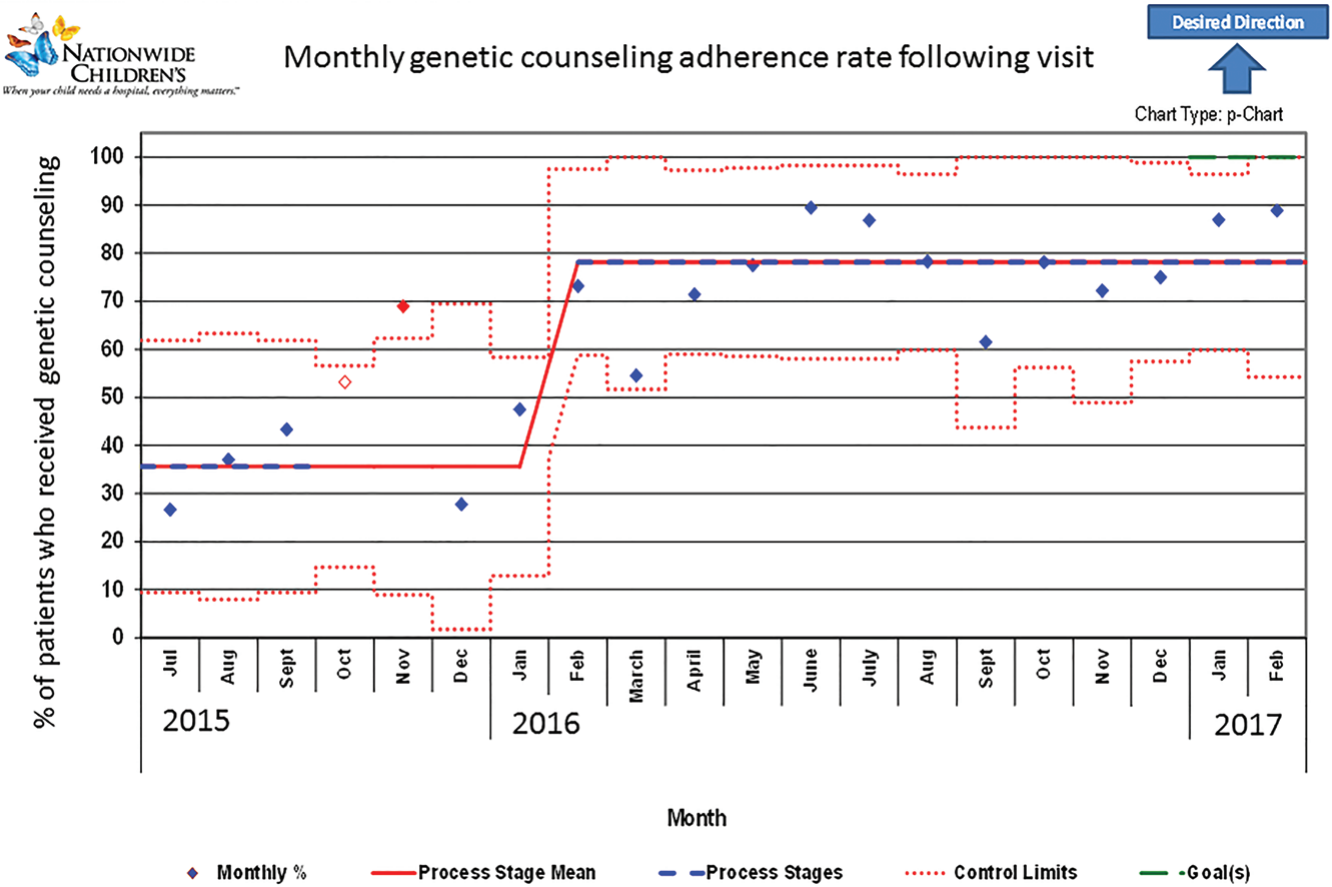
Monthly genetic counseling adherence rate following a visit to a multidisciplinary clinic for Down syndrome; geneticist presence implemented in October 2015 increases adherence.

Due to genetic counseling adherence rate being lower than chromosome adherence rate in our baseline sample, we focused on this aspect in additional detail. In addition to analyzing data for all patients, our monthly genetic counseling adherence (number of adherent patients in month/total number of patients in month) was tracked over time (Fig. 1). This p-chart demonstrated a shift in adherence in April 2016. Median baseline monthly adherence to genetic counseling before arrival was 35% before April 2016; the median adherence after April 2016 was 62%. This difference is significant, *P* < 0.001 (Fisher’s exact test) and sustained through the end of the study period with a final monthly adherence of 82% in February 2017. Calculating postvisit rates of genetic counseling adherence showed similar patterns (Fig. 2). Baseline adherence rate of 36% showed improvement beginning in November coinciding with Genetics intervention in mid-October leading to shift in February and resulting in a postintervention adherence rate of 78%. Genetic counseling adherence rate continued to improve with a final monthly adherence of 89% in February 2017.

Subanalysis by age showed similar trends. The baseline genetic counseling adherence rate at arrival of 38% for those less than 12 months of age did not differ from the remainder of the sample, *P* = 0.08. Genetic counseling adherence rates improved in all age ranges with improvement of statistical significance when comparing baseline and postintervention rates (Table 3).

**Table 3.**
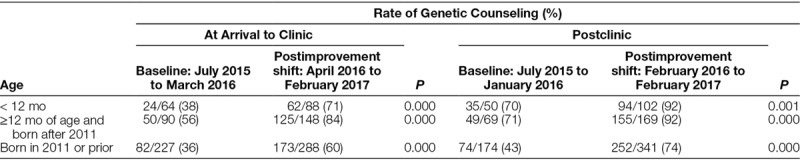
Age-Related Genetic Counseling Adherence for Down syndrome and Improvement with Implementation of a Clinical Geneticist

**Fig. 3.**
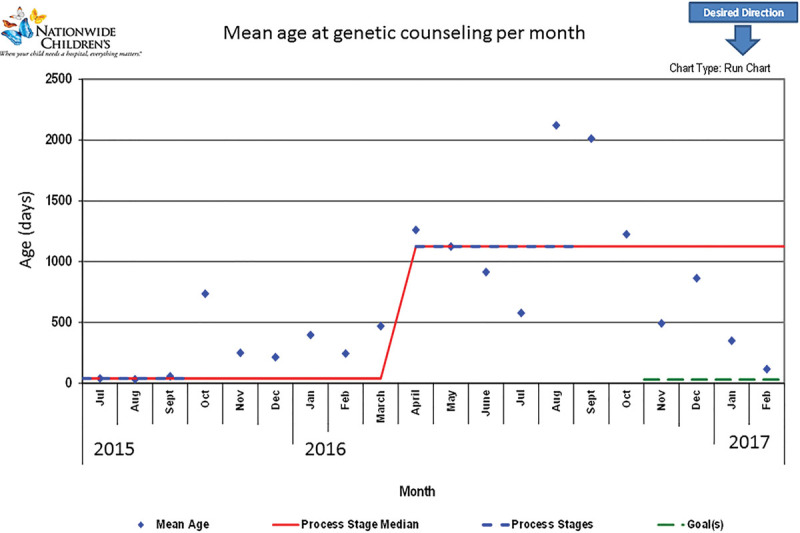
Mean age at genetic counseling in Down syndrome per month; geneticist presence implemented in October 2015 increases age due to “catch-up” genetic counseling.

During our study period, we tracked the age of the child with Down syndrome at the time of genetic counseling. Before geneticist presence in the clinic in October 2015, the median baseline age at genetic counseling was 39 days. Plotting age at genetic counseling in a run chart showed a shift in age in April 2016; after which the median age was 3.1 years (Fig. 3). This increase in age was due to catch-up counseling for older children whose parents had never previously received genetic counseling and showed trends in decreasing age at the end of the study.

## DISCUSSION

Genetic counseling can have positive impacts on patient communication and education as well as improve adherence to medical management recommendations.^11,12^ The American Academy of Pediatrics and others have published guidelines for care of children with Down syndrome for more than 30 years.^6,13,14^ Genetic counseling and chromosome analysis are some of the first recommendations in the care of children with Down syndrome.^6^ Genetic counseling for parents of a child with Down syndrome is essential as a means of education, parent support, and providing ongoing information through the lifespan.^15^

Prior studies have found low levels of adherence with the published guidelines.^16–18^ Unfortunately, publication alone does not translate into increased adherence, but subspecialist involvement can improve care.^19–21^ Previously, pediatrician education led to increased adherence to select guidelines for Down syndrome, including referrals to Genetics.^8^ No quality improvement studies for Down syndrome have focused specifically on genetic counseling in a subspecialty clinic for Down syndrome.

Baseline adherence to genetic counseling at Nationwide Children’s Hospital was similar to previous studies and in need of improvement.^8^ As expected, direct geneticist presence significantly improved genetic counseling adherence rates to 62%, *P* < 0.001. Postclinic genetic counseling adherence and age-related statistical analyses showed improvement. Strengths of this project include the focus on genetic counseling and chromosome studies, unaddressed in a dedicated way before this project, with improved adherence to these measures.

Although adherence to genetic counseling was low and showed significant improvement, we did not investigate the specific cause for poor adherence. Parent survey has previously shown that parents of children with Down syndrome do not understand the role of a genetic counselor before their visit with one.^22^ Education to gain buy-in from stakeholders may continue to improve adherence. Additionally, adherence may be impacted by including all scheduled patients; this could underestimate adherence. Long wait times, delays in ordering referral, or limited availability of geneticists could contribute to the delay in genetic counseling. Lack of communication, both within an institution and among various institutions, and differences in EMR documentation without a centralized process are other potential causes for missed opportunities for genetic counseling.

Geneticist involvement increased documentation of chromosome studies and rarely led to increased chromosome laboratory ordering. Overall, chromosomes were often completed but not readily available in the current EMR. Some factors that could contribute to this include chromosomes ordered through a different hospital system, chromosomes ordered under a different name (infants are routinely listed with mother’s last name), and amniocentesis ordered under a mother’s name. As this was not the primary focus of our study, we did not investigate this specifically, but found that 29% of patients had chromosomes completed that were not easily found in the internal EMR. Chromosome study results not documented in the EMR was a significant challenge. Documentation is essential in preventing pediatricians from repeating chromosome studies unnecessarily and allowing genetics professionals to find the information needed to provide accurate genetic counseling. In 1 instance, a translocation (21;21) not previously documented in the patient’s chart was identified through this initiative. In this case, the translocation was de novo, but had the potential for inheritance from a parent with a balanced Robertsonian translocation significantly changing the recurrence risk. A geneticist added value through improved documentation, communication, and avoidance of duplicative ordering.

Beyond the process of genetic counseling and uploaded chromosome reports, the presence of a geneticist assisted in answering numerous parental questions and in improving parental understanding of genetics. Genetic counseling should be performed in a timely manner to maximize parental education and uptake. Age at initial genetic counseling increased following geneticist involvement in the clinic: from approximately 1 month to 3 years. Unfortunately, many patients in the Down syndrome clinic had not received appropriate counseling in the newborn period; “catch-up” genetic counseling was completed in many children > 1 year of age. This is not ideal or the standard of care; however, genetic counseling at any point continues to show benefits.^3,7^ With a continued genetics presence in the clinic, we anticipate that the age at genetics visit will decrease over time with the aim that all infants meet with a geneticist by 1 month of age.

Our study focused on all patients seen in a subspecialty clinic for Down syndrome; this may limit the generalizability of our results due to selection bias. However, our initial adherence rate was similar to a published rate from review of outpatient pediatrics clinics, suggesting that our clinic population is similar to a general outpatient pediatric clinic population.^8^ Patients seeking care in this subspecialty clinic might be more connected with medical resources and more likely to have seen a genetics professional; this would cause our results to overestimate the adherence rate suggesting an even greater need for improvement. By including patients who did not keep their appointment, we attempted to improve generalizability and include those patients who might be more likely to not keep a genetics clinic appointment. The number of referrals to genetics that were not completed was low at 4%; parent follow-through is not the primary barrier to genetic counseling. Using adherence to genetic counseling as our outcome measure may be restrictive and fail to capture genetic counseling completed by nongenetics professionals but was the most consistent, measurable outcome available. Additional limitations could include inaccessibility of medical documentation at outside institutions, parent recall of receiving genetic counseling, and changes in medical documentation from paper notes to EMRs.

Ongoing, expanding quality improvement efforts aim to improve the timing of genetic counseling for Down syndrome. Implementing our improvement strategy at other institutions requires the availability of a genetics professional that may be limited due to staffing, cost, and other factors. Future studies could address translation and determine if our model is sustainable. Sustainability requires that the value of genetics professionals is appreciated and that this position remains maintained over time. Although not universal, multidisciplinary models including genetics professionals exist. Of the 66 Down syndrome specialty clinics in the United States, 41 include a genetics professional.^23^ However, it is unclear how these clinic models compare to ours and if some might have an “on-call genetic counselor” as Nationwide Children’s Hospital did prior to this project. Alternatively, if a genetics professional is not available, another improvement model could focus on the education of developmental pediatricians or others to ensure discussion of genetic concepts occurs. This requires time, commitment to collect laboratory reports from outside institutions, communication with the cytogenetics laboratory, and up-to-date information about current genetic testing methodologies such as cell-free fetal DNA. With the many other nongenetic issues to address in a clinical visit, lengthier appointments are likely necessary to allow time for genetic counseling. Future studies could continue to focus on the underlying cause for nonadherence, how nonadherence influences families’ experiences at diagnosis, and the utility of multidisciplinary care to address other guidelines for care.

## CONCLUSIONS

Many children with Down syndrome had not met with genetics before arrival at the outpatient Down syndrome clinic at Nationwide Children’s Hospital. Genetic counseling is an important component of care in newborns with Down syndrome warranting continued improvement. Multidisciplinary clinics for Down syndrome should integrate geneticists and genetic counselors to ensure the correct genetic testing has been completed, is documented in the patient’s records, and families receive appropriate genetic counseling.

## ACKNOWLEDGMENTS

Appreciation is given to members of the multidisciplinary Down syndrome clinic for their willingness to integrate genetics into the clinic.

## DISCLOSURE

The authors have no financial interest to declare in relation to the content of this article.

## Supplementary Material
