# Supplementary material for: Integrating a Geneticist in a Multidisciplinary Clinic for Down Syndrome Increases Commitment to Genetic Counseling
Source: Pediatr Qual Saf. 2017 Aug 25;2(5):e039. doi: 10.1097/pq9.0000000000000039 (PMC6132463; doi:10.1097/pq9.0000000000000039)
Supplement: Supplementary file 1 [file pqs-2-e039-s001.pdf]

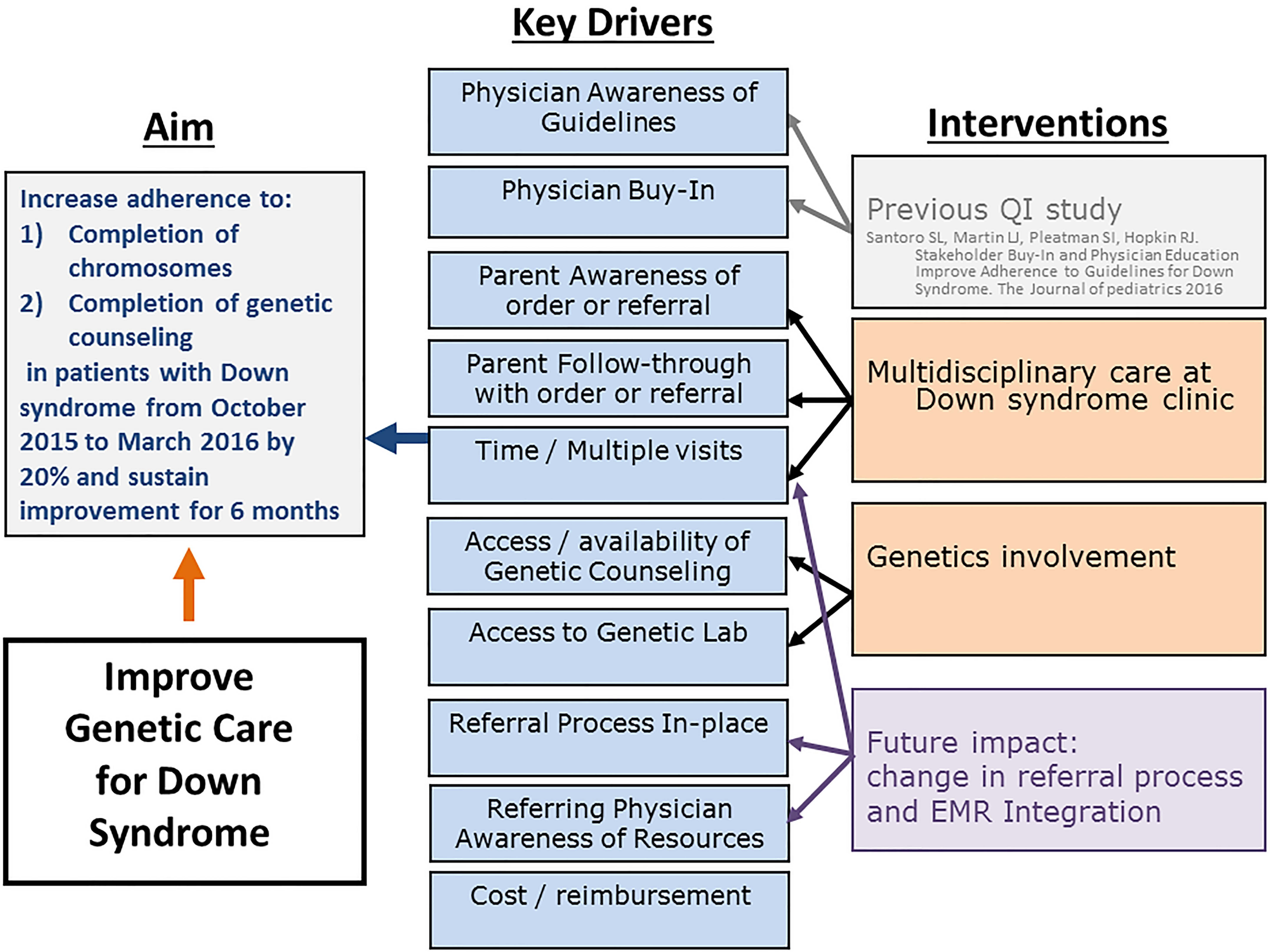
Key Drivers
Aim
Interventions
Increase adherence to:
1) Completion of chromosomes
2) Completion of genetic counseling
in patients with Down syndrome from October 2015 to March 2016 by 20% and sustain improvement for 6 months
Improve Genetic Care for Down Syndrome
Physician Awareness of Guidelines
Physician Buy-In
Parent Awareness of order or referral
Parent Follow-through with order or referral
Time / Multiple visits
Access / availability of Genetic Counseling
Access to Genetic Lab
Referral Process In-place
Referring Physician Awareness of Resources
Cost / reimbursement
Previous QI study
Santoro SL, Martin LJ, Pleatman SI, Hopkin RJ. Stakeholder Buy-In and Physician Education Improve Adherence to Guidelines for Down Syndrome. The Journal of pediatrics 2016
Multidisciplinary care at Down syndrome clinic
Genetics involvement
Future impact: change in referral process and EMR Integration
